# Alexithymia and sensory processing sensitivity account for unique variance in the prediction of emotional contagion and empathy

**DOI:** 10.3389/fpsyg.2023.1072783

**Published:** 2023-04-20

**Authors:** Amanda M. McQuarrie, Stephen D. Smith, Lorna S. Jakobson

**Affiliations:** ^1^Department of Psychology, University of Manitoba, Winnipeg, MB, Canada; ^2^Department of Psychology, University of Winnipeg, Winnipeg, MB, Canada

**Keywords:** empathy, emotional contagion, alexithymia, sensory processing sensitivity (SPS), individual differences, childhood emotional abuse, personality variables

## Abstract

**Introduction:**

Empathy—the ability to identify and share another person’s emotional state—is an important socio-emotional process arising, in part, from emotional contagion. In the current study, we assessed unique variance in emotional contagion and other empathy-related constructs accounted for by two personality traits, alexithymia and sensory processing sensitivity (SPS), when controlling for childhood emotional abuse and current depressed mood.

**Methods:**

A sample of 305 adults (*M*_*age*_ = 20.1 years) watched brief film clips chosen to induce various emotional states. After each film, the participants rated how strongly they experienced each of nine different emotions. They then completed self-report measures of alexithymia, SPS, empathy-related constructs, childhood emotional abuse, and current mood.

**Results:**

Those scoring high (vs. low) on SPS reported stronger primary emotions and a larger range of emotions when watching the films and were more apt to believe that their emotions matched those of the individuals featured in the films. They also scored higher on both self-oriented processes (such as the tendency to feel personal distress in tense situations) and other-oriented processes (such as perspective taking and empathic concern) related to empathy. Individuals scoring high (vs. low) on alexithymia reported feeling a larger range of emotions while watching the films but scored lower on other-oriented processes related to empathy. After controlling for SPS and alexithymia, current depressed mood predicted experiencing less varied reactions to mixed valence films that elicited strong feelings of embarrassment/humiliation, and less amusement when watching positive films. Childhood emotional abuse did not emerge as a predictor of emotional contagion or empathy.

**Discussion:**

We propose that the strong and nuanced feelings elicited in those scoring high on SPS by observing others support their personal view that they are highly empathic. In contrast, by failing to closely examine their own mixed reactions to others, individuals with alexithymia may find it difficult to connect with, understand, and respond to others’ feelings.

## 1. Introduction

Empathy has proven to be difficult to define; in fact, a review by Cuff et al. (2016) found 43 distinct definitions of empathy from various authors. Based on their review, these authors defined empathy as the process of identifying and sharing another person’s emotional state. The important component of this definition is that it captures both affective processes (experiencing the same emotion as another) and cognitive processes (recognizing that the emotion one is feeling matches that of another). Although there is evidence that these processes activate different brain regions, they are not separate from one another; rather, they are complementary components that influence one another to produce the overarching construct of empathy (Cuff et al., 2016). Both processes are underpinned by emotional contagion—an automatic, affective process that stems from the automatic mimicry of others’ movements (e.g., facial expressions, body postures, or gaze) and autonomic responses (e.g., heart rate, pupillary changes, blushing; Prochazkova and Kret, 2017; Zurek and Scheithauer, 2017). Although we may not be fully aware of all aspects of this response, those aspects that reach awareness are embraced and experienced fully by the individual; they underlie our understanding of how *we* feel (Bird and Viding, 2014). Top-down control is required to effect the switch from self- to other-oriented processing needed to support the transition from emotional contagion to full empathy (Bird and Viding, 2014).

The term empathy is often used interchangeably with other terms such as theory of mind, compassion, sympathy, and perspective-taking, despite differences in the information gained by the particular process and the activated neural regions (Bird and Viding, 2014). This has led to inconsistencies in how empathy is measured, making cross-study comparisons difficult (Gerdes et al., 2010). Indeed, self-reported empathy does not always predict performance on behavioral measures of empathy (Murphy and Lilienfeld, 2019). Thus, although it is common practice to utilize self-report measures to study these constructs (Gerdes et al., 2010; Ilgunaite et al., 2017), it is useful to collect both types of measures.

To explore links between self-report measures and behavior, Jordan et al. (2016) had participants complete a range of behavioral measures assessing prosocial action tendencies, along with two questionnaires assessing empathy and related constructs: the Interpersonal Reactivity Index (IRI; Davis, 1983) and the Empathy Index (EI; Jordan et al., 2016). The IRI includes four subscales that tap into the tendency to feel anxiety or unease in tense interpersonal settings (Personal Distress or PD), spontaneously adopt another’s point of view (Perspective-taking or PT), feel concern for those in need (Empathic Concern or EC), and imagine yourself in fictional situations (Fantasy or FS). The EI subscales assess one’s awareness of how closely one’s own emotional responses mimic those of others (Empathy or EMP) and how closely one’s own actions mimic those of others (Behavioral Contagion or BC). Jordan et al. (2016) reported that the six subscales of the combined IRI/EI loaded onto two factors. Factor 1 included the PD, EMP, and BC subscales, all of which include items requiring the respondent to reflect on their *own* distress, emotions, or behaviors—processes that are largely self-oriented. Factor 2 included the PT and EC subscales, both of which focus on other-oriented processes. Items from the FS subscale loaded similarly on both factors. Importantly, Jordan et al. (2016) found that feeling *concern for* an empathy target (i.e., scoring high on the other-oriented factor) was a more important predictor of altruistic giving than *feeling like* that target (i.e., scoring high on the self-oriented factor). The authors suggested that this may be especially true when the empathy target is experiencing considerable distress as this would create a strong sense of *personal* distress in those scoring high in contagion/empathy, which could be somewhat debilitating for action.

### 1.1. Individual differences in empathy and related constructs

Experiencing atypical levels of empathy (as seen in certain psychiatric conditions) can contribute to interpersonal difficulties or distress (e.g., Decety and Moriguchi, 2007; Zurek and Scheithauer, 2017). As such, it is important to investigate factors that underlie individual differences in empathy and related constructs. In this regard, it is likely that both genetic/epigenetic factors (including personality traits with a genetic component) and experiential (learning-related) factors play a role in shaping these important processes.

One personality trait that has been a focus of research in this area is alexithymia. Alexithymia is characterized by difficulties identifying and expressing one’s feelings, and by a cognitive style that is externally oriented and concrete (Sifneos, 1973). Hogeveen and Grafman (2021) describe the emotion-processing difficulties experienced in alexithymia as reflecting a disconnect between receiving an arousal signal and understanding that signal. Alexithymia has also been related to a deficit in interoceptive processing (i.e., difficulties separating interoceptive states like nausea from emotions; Brewer et al., 2016); however, some evidence suggests that problems with interoceptive accuracy may only characterize individuals with one subtype of alexithymia (Jakobson and Rigby, 2021).

There are both developmental and acquired forms of alexithymia, the latter being a feature of several neurological disorders (Hogeveen and Grafman, 2021). Our focus was on the developmental form. It has a clear genetic component (Mezzavilla et al., 2015); indeed, even after covarying out depression (which is correlated with alexithymia), heritability is estimated to be 33% (Picardi et al., 2011). However, environmental influences also play a large role in the expression of developmental alexithymia. For example, there is an increased prevalence of this form of alexithymia in individuals who have experienced trauma or adverse environments growing up (Kopera et al., 2020). Karaca Dinç et al. (2021) found that alexithymia mediates the relationship between childhood trauma and depression, anxiety, and negative self-esteem. They suggested that trauma impairs an individual’s ability to process arousal signals and to identify or differentiate between conscious emotions, leading to difficulties with regulating emotions and pathological negative affectivity (Thoma et al., 2011; Banzhaf et al., 2018).

Findings from neuroimaging studies suggest that alexithymia is associated with decreased activity in neural regions associated with empathy, self-other perception, emotional resonance, and emotional expression (e.g., Moriguchi et al., 2007). Individuals with alexithymia also generally report low levels of empathy (e.g., Aslan et al., 2020). Although results are somewhat mixed (e.g., Beadle et al., 2013; MacDonald and Price, 2017), impairments in both other-oriented processes [such as the ability to identify the emotional state of another person (e.g., Grynberg et al., 2010; Mul et al., 2018; Brett and Maybery, 2022)] and self-oriented processes (e.g., Goerlich-Dobre et al., 2015) have been described. The self-oriented process that has been most consistently related to alexithymia is the tendency to experience increased levels of personal distress and discomfort in response to distress in others (e.g., Guttman and Laporte, 2002; Moriguchi et al., 2007; Beadle et al., 2013; Patil and Silani, 2014; Banzhaf et al., 2018; Brewer et al., 2019; Di Tella et al., 2020). This heightened response may arise from atypicalities in emotional contagion, problems with emotion regulation, and/or difficulties creating the self-other separation required to reduce personal distress during empathy (Moriguchi et al., 2007; Brewer et al., 2019).

Another personality trait that may be relevant to the study of empathy is sensory processing sensitivity (SPS). SPS is a hereditary personality trait characterized by the deeper cognitive processing of physical, social, and emotional stimuli, as well as by a nervous system that is easily overwhelmed by environmental stimuli and is more sensitive to subtleties (e.g., aesthetic features of the environment), pain, others’ moods, and the arts (Aron and Aron, 1997; Aron et al., 2012). The prevalence of this trait is between 15 and 30% in the general population (Aron and Aron, 1997; Lionetti et al., 2018). Aron et al. (2005) found that experiencing an adverse environment in childhood impacts individuals with SPS to a greater extent than those without SPS, often leading to increased negative affectivity (including fearfulness, anxiety, and depression) in adulthood (see also Aron et al., 2012; Acevedo, 2020). Karaca Dinç et al. (2021) found that SPS mediated the positive relationship between childhood trauma and increased negative affectivity, such that childhood trauma positively predicted SPS, which positively predicted negative affectivity.

Research investigating relationships between SPS and specific measures of empathy and related constructs is limited; however, neuroimaging research suggests that individuals scoring high on this trait show increased activity in regions associated with empathy and self-other awareness (e.g., inferior frontal gyrus and insula) and self-referential processing (e.g., temporoparietal junction) during the viewing of emotional pictures (Acevedo et al., 2018). These findings suggest that SPS may be positively associated with empathy, which one might expect given that this trait is characterized by introspection, heightened emotional awareness, and increased sensitivity to others’ emotional states. Indeed, Schaefer et al. (2022) reported positive associations between SPS and all subscales of the IRI, with the exception of PT.

### 1.2. The current study

Although alexithymia and SPS appear to differ in several ways, they are related; thus, difficulties identifying and describing feelings have been found to be positively correlated with sensory sensitivity (ease of excitation and low sensory threshold), and externally oriented thinking has been found to be negatively correlated with aesthetic sensitivity (Liss et al., 2008; Jakobson and Rigby, 2021). Alexithymia and SPS have also been found to co-occur in a subset of the population (Jakobson and Rigby, 2021; Karaca Dinç et al., 2021). Despite these findings, however, few authors have investigated these traits in the same study, while also accounting for the impacts of early adversity and depression. The overarching goal of the current study was to use this approach in order to tease apart the *unique contributions* that these traits make to the prediction of both self- and other-oriented processes related to empathy.

The findings from Jordan et al. (2016), discussed earlier, highlight the importance of supplementing self-report measures with behavioral measures in research of this kind. In the current study, we administered a behavioral task designed to allow for the measurement of emotional contagion, which underlies many empathy-related processes. Specifically, the task involved showing individuals emotional films and then asking them to identify both the emotion(s) that they personally experienced (explicit/conscious emotional contagion), and the extent to which they believed that their own emotional response(s) were similar to those of the empathy target (self-other matching). We then went on to assess the *unique contributions* that alexithymia and SPS make to the prediction of different facets of the contagion response, when holding effects related to early adversity and depression constant.

It was hypothesized that, when holding other potentially relevant variables constant, alexithymia and SPS would show different relationships to both the conscious experience of emotional contagion and to empathy and related constructs assessed through self-report. Specifically, we predicted that: (a) scoring high (vs. low) on alexithymia would be associated with experiencing weaker and/or more mixed reactions to the film clips and with deficits in other-oriented processes; and (b) that scoring high (vs. low) on SPS would be associated with reacting more strongly to the film clips and with strengths in both self- and other-oriented processes. In addition to the above, we predicted that features of participants’ conscious, emotional reactions to the films would predict ratings of empathy and related constructs.

## 2. Materials and methods

### 2.1. Participants

Adult participants were recruited from the University of Manitoba. Participants were Canadian citizens, reported English as their first language, and reported no history of neurological disorders or significant head injuries. They received course credit toward an optional research participation component for participating in the study. After cleaning the data (see below), we were left with a final sample of *N* = 305. This sample size was more than adequate based on *a priori* sample size calculation for multiple regression. The mean age of participants was 20.1 years (SD = 4.7; range = 17–44). Given that 81.3% of participants reported their sex as female, sex differences were not explored in the present study. All participants provided informed, written consent prior to participation. Ethical approval was received from the Research Ethics Board at the University of Manitoba (Fort Garry campus). The study was not pre-registered.

### 2.2. Measures

#### 2.2.1. Toronto Alexithymia Scale—20 items

The Toronto Alexithymia Scale (TAS-20) is a 20-item self-report measure of alexithymia that taps into three key features: difficulties identifying feelings, difficulties describing feelings, and externally oriented thinking (Bagby et al., 1994a). Items are answered using a five-option Likert scale ranging from 1 (*strongly disagree*) to 5 (*strongly agree*). Five items are reversed-scored. Item scores are summed to produce a total score that can range from 20 to 100. The TAS-20 is a reliable and valid measure (Bagby et al., 1994a,b). In this study, the TAS-20 possessed high reliability, with a Cronbach’s α = 0.86.

#### 2.2.2. Highly Sensitive Person Scale

The Highly Sensitive Person Scale (HSPS) is a 27-item self-report measure of SPS that taps into three key features of SPS: ease of excitation, low sensory threshold, and aesthetic sensitivity (Aron and Aron, 1997). Each item is rated on a seven-option Likert scale from 1 (*not at all*) to 7 (*extremely*). The measure is scored by calculating the mean (range 1 to 7). The HSPS possesses strong reliability and validity (Aron and Aron, 1997; Smith et al., 2019). In the current study, this measure was also found to have strong reliability, with a Cronbach’s α = 0.89.

#### 2.2.3. Adult Temperament Questionnaire—Orienting Sensitivity subscale

Aron et al. (2012) have recommended that the Orienting Sensitivity subscale of the Adult Temperament Questionnaire (OS-ATQ; Evans and Rothbart, 2007) be administered along with the HSPS to assess the full range of features associated with SPS. Total scores on the OS-ATQ and the HSPS are strongly correlated (*r* = 0.63; Evans and Rothbart, 2008), suggesting that the constructs are closely related. The OS-ATQ is a 15-item self-report measure that assesses one’s level of emotional and cognitive awareness of internal and external stimuli; however, it also taps into processes that are not driven by sensory stimuli, such as aspects of problem solving and imagery, which are related to SPS but not specifically assessed by the HSPS. Each item is rated on a seven-point Likert scale from 1 (*extremely untrue*) to 7 (*extremely true*), with an X (*not applicable*) option. Four items are reverse scored. Item values are averaged to produce a total score (range 1 to 7). Internal consistency for this measure in the current study was reasonably strong, with a Cronbach’s α = 0.73.

#### 2.2.4. Interpersonal Reactivity Index and Empathy Index

The combined IRI/EI is a 42-item self-report measure that, as noted earlier, includes the four subscales that comprise the IRI (Davis, 1983; PT, PD, EC, FS) along with two additional subscales that comprise the EI (Jordan et al., 2016; EMP, BC). Each item is rated on a four-option Likert scale, from 0 (*does not describe me well*) to 4 (*describes me very well*). Nine items are reverse scored. Each subscale is calculated by averaging item scores (range 0 to 4). In the current study, Cronbach’s α was 0.86 for the total measure, indicating strong internal consistency. Reliability analyses for all subscales except the BC subscale produced an acceptable Cronbach’s α ≥ 0.60. After removing two items from the BC subscale, all Cronbach’s α values fell within the acceptable range. Therefore, in the current analyses, the BC subscale score was based on five items, whereas each of the other subscale scores were based on seven items.

#### 2.2.5. Childhood Trauma Questionnaire (CTQ) short form—Emotional Abuse subscale

This self-report measure assesses the level of emotional abuse one experienced throughout childhood (Bernstein et al., 2003). Individuals are asked to think about experiences they had as a child or teenager and to rate their agreement with each item on a five-option Likert scale from 1 (*never true*) to 5 (*very often true*). Five items are included in this subscale. Item scores are summed to produce a total emotional abuse subscale score (range 5 to 25). This measure has strong validity and reliability (Bernstein et al., 2003). In the current study, the emotional abuse subscale possessed strong internal reliability, with a Cronbach’s α = 0.91.

#### 2.2.6. Patient Health Questionnaire—9 items

The Patient Health Questionnaire-9 (PHQ-9) is a nine-item self-report depression screening measure with strong validity (Kroenke et al., 2001). When completing this measure, individuals rate the frequency of their experience of different symptoms of depression over the past 2 weeks from 0 (*not at all*) to 3 (*nearly every day*). The items are summed to produce a total score that can range from 0 to 27. In this study, the PHQ-9 possessed strong internal reliability, with a Cronbach’s α = 0.88.

### 2.3. Procedure

This study was administered online using Qualtrics Online Survey Software. Participants provided informed consent, answered demographic questions regarding their sex and age, completed the behavioral task that was designed to elicit emotional contagion, and then answered the previously described self-report measures of alexithymia, SPS, empathy and its related constructs, childhood emotional abuse, and current depressive symptoms.

During the behavioral task, participants watched 10 different film clips chosen from a collection compiled by Samson et al. (2016) that have been shown to reliably induce different levels of positive (amusement, pride, and love), negative (repulsion, fear, sadness, and anger), and/or mixed emotions (brief descriptions of the clips are provided in the Supplementary material). Each clip was approximately 30 s in length and did not include audio. The clips were natural and realistic and had not been professionally created or edited. There were two positive clips (“positive”), two mixed-valenced clips that showed someone in an embarrassing situation that others might find amusing (“mixed/embarrassing”), two mixed-valence clips that showed someone in an embarrassing situation that others might find humiliating (“mixed/humiliating”), two negative clips (“negative”), and two neutral clips (“neutral”). One clip of each type showcased a single individual, and the other featured an individual who was part of a large group.

Before watching each clip, participants were instructed to “direct [their] whole attention to the film, let the film sink in and try to feel with the person in the film.” After watching the clip, participants were instructed to indicate how strongly they agreed that they had *personally* experienced each of nine different emotions (amusement, pride, love, repulsion, fear, sadness, anger, boredom, and embarrassment) using a six-option Likert scale ranging from 1 (*do not agree at all*) to 6 (*very strongly agree*). Participants were explicitly asked to use a rating of 1 (*do not agree at all*) if they felt that they had not experienced a given emotion. This instruction was included to address the increased tendency for participants with alexithymia to report not experiencing any emotion (Aaron et al., 2018). Finally, participants were asked to rate how closely they thought their “feelings while watching the film clip matched those experienced by the main person in the clip” on a six-option Likert scale from 1 (*very different*) to 6 (*very similar*). This question was included to tap into other-oriented processes required to identify what the individual featured in each film clip was feeling and reflect on the match. Ratings in the behavioral task were averaged across the two videos of each type (individual vs. group context).

Two steps were taken to minimize the likelihood of carry-over effects while watching the film clips. First, after viewing each video clip and responding to the associated questions, participants completed two items chosen from the Edinburgh Handedness Inventory (Oldfield, 1971) before proceeding to the next video. In completing this inventory, individuals are simply asked to indicate which hand they prefer to use when completing a variety of unimanual acts—a task that is low in cognitive and emotional demands. Responses to these items were not analyzed. Second, we alternated between a positive or neutral clip and a negative or mixed valence clip to avoid having several clips of the same valence occur in sequence. The exception was that the series always ended with two positive/neutral clips in order to minimize the chance that participants would finish the task in a negative emotional state.

### 2.4. Data cleaning and imputation of missing data

A total of 340 individuals completed the online study. Prior to inferential testing, data were cleaned by removing duplicate responses; checking for proper coding of variables; and removing participants who did not complete at least one subscale of any questionnaire, did not complete ratings for one (or more) video(s) in the behavioral task, and/or took less than 5 min or more than 2 h to complete the study. Five minutes was used as the lower cutoff for time-to-completion as it took approximately 5 min to simply view the video clips; 2 h was set as the upper cut-off due to the possibility that the participants’ emotional state could have significantly changed (due to external influences) over that length of time. Thirty-five participants were removed during data cleaning, leaving a final sample of *N* = 305.

After cleaning the data, Little’s Missing Completely at Random (MCAR) test was run and confirmed that missing data were MCAR. Less than 0.005% of values were missing, and these were imputed using an Estimation-Maximization algorithm. Outliers were identified and corrected through winsorizing, dependent variables were checked for normality, and linearity between independent and dependent variables was confirmed. These analyses, and the descriptive and inferential statistical analyses described below, were conducted using IBM SPSS Statistics for Windows, Version 28. Unless otherwise indicated, an alpha of 0.05 was assumed as the basis for statistical significance.

## 3. Results

### 3.1. Statistical analyses

The section “3. Results” begins with an examination of the zero-order correlations between total scores on the different self-report measures. The Benjamini-Hochberg procedure was used with a False Discovery Rate (FDR) of 0.05 to control for the increased probability of Type 1 errors when testing multiple correlations. This confirmed that the variables related to one another in ways that were consistent with past research. Following this, we summarize findings from two analysis of variance (ANOVA) tests that allowed us to characterize participants’ emotional reactions to the five different types of films. Greenhouse-Geisser adjustments were made to the degrees of freedom where appropriate.

Next, we present the results of a series of multivariate multiple regression (MMR) analyses that allowed us to assess unique variance in performance on our behavioral task, and in empathy-related processes assessed through self-report, that was accounted for by SPS, alexithymia, childhood emotional abuse, and depression. In all MMR analyses, we identified significant path coefficients by examining bias-corrected accelerated 95% confidence intervals calculated using 1000 bootstrapped samples. In these MMR analyses, the covariance between predictor variances and between outcome variables were taken into account.

An exploratory factor analysis (EFA) was conducted on the IRI/EI subscales. The data were first examined for appropriateness, and it was determined that the data met the requirements for EFA. The principal axis factoring extraction method and oblique promax rotation method were used, mirroring the approach taken in the EFA completed by Jordan et al. (2016).

Finally, we report correlations between measures extracted in the behavioral task and both self- and other-oriented processes related to empathy assessed through self-report. Again, the Benjamini-Hochberg procedure was applied with an FDR of 0.05 to control for the increased probability of Type 1 errors when conducting multiple correlations.

### 3.2. Zero-order correlations between study measures

As seen in Table 1, HSPS scores were positively correlated with both the TAS-20 scores and the OS-ATQ scores with small-to-medium effect sizes, and TAS-20 and OS-ATQ scores were not significantly correlated. All three personality variables were positively correlated with both the CTQ and PHQ-9 scores with small-to-medium effect sizes, and the latter two variables were also positively correlated with one another with a medium effect size. As correlations between the variables listed above were in the small-to-medium range, these associations were not strong enough to create problems related to multicollinearity in the analyses described below.

**TABLE 1:** Pearson correlation coefficients measuring the relationships between the study measures.

	TAS-20	HSPS	OS-ATQ	EC	PT	FS	PD	EMP	BC	CTQ
TAS-20	–									
HSPS	0.27**	–								
OS-ATQ	−0.09	0.43**	–							
EC	−0.13*	0.29**	0.34**	–						
PT	−0.24**	0.17**	0.29**	0.43**	–					
FS	−0.03	0.37**	0.40**	0.31**	0.19**	–				
PD	0.44**	0.46**	−0.01	0.18**	−0.03	0.05	–			
EMP	0.14*	0.53**	0.38**	0.48**	0.27**	0.37**	0.38**	–		
BC	0.08	0.38**	0.27**	0.31**	0.13*	0.37**	0.20**	0.54**	–	
CTQ	0.24**	0.25**	0.13*	−0.05	0.03	0.07	0.17**	0.08	0.12*	–
PHQ-9	0.49**	0.42**	0.18**	−0.03	−0.06	0.12	0.29**	0.19**	0.18**	0.43**

TAS-20, Toronto Alexithymia Scale; HSPS, Highly Sensitive Person Scale; OS-ATQ, Orienting Sensitivity subscale from the Adult Temperament Questionnaire; CTQ, Emotional Abuse subscale of the Childhood Trauma Questionnaire; PHQ-9, Patient Health Questionnaire. SubScales of the Interpersonal Reactivity Index/Empathy Index: EC, Empathic Concern; PT, Perspective-taking; FS, Fantasy; PD, Personal Distress; EMP, Empathy; BC, Behavioral Contagion.

*Correlation is significant at the 0.05 level (2-tailed).

**Correlation is significant at the 0.01 level (2-tailed).

Most of the IRI/EI subscales were correlated with each other, with the exception of the PT and PD subscales and the FS and PD subscales. The HSPS and OS-ATQ total scores were positively correlated with all the IRI/EI subscales, except for a non-significant negative correlation between the OS-ATQ and the PD subscale. This supports the view that those with SPS tend to believe themselves to be generally empathic. In contrast, those scoring higher on alexithymia reported being more aware that others’ emotions affected them *personally* (positive correlations with PD and EMP), but also reported a weakness in understanding others’ perspectives or feeling concern for them (negative correlations with PT and EC); in other words, they reported being more self-oriented than other-oriented. Interestingly, CTQ scores were positively correlated with the PD and BC subscale scores, and PHQ-9 scores were positively correlated with the PD, EMP, and BC subscale scores; thus, both childhood emotional abuse and current depressive symptoms were also more closely related to self-oriented processes than to other-oriented processes.

The fact that both CTQ and PHQ-9 scores were associated with scores on a range of other study variables highlights the importance of controlling for past emotional abuse and current mood when assessing links between personality variables and empathy-related constructs.

### 3.3. Contagion profiles and identification of the “primary” emotion elicited by each type of film

The 5 (Film Type) X 9 (Emotion Rating) ANOVA conducted on ratings of emotions experienced when viewing the films confirmed that mean ratings differed significantly both across film types, *F*_(3.32, 1008.35)_ = 203.94, *p* < 0.001, *η_*p*_*^2^ = 0.40, and across emotions, *F*_(5.08, 1544.42)_ = 265.31, *p* < 0.001, *η_*p*_*^2^ = 0.47. There was also a significant interaction effect between film type and emotions, *F*_(12.97, 3941.54)_ = 440.52, *p* < 0.001, *η_*p*_*^2^ = 0.59.

**FIGURE 1:**
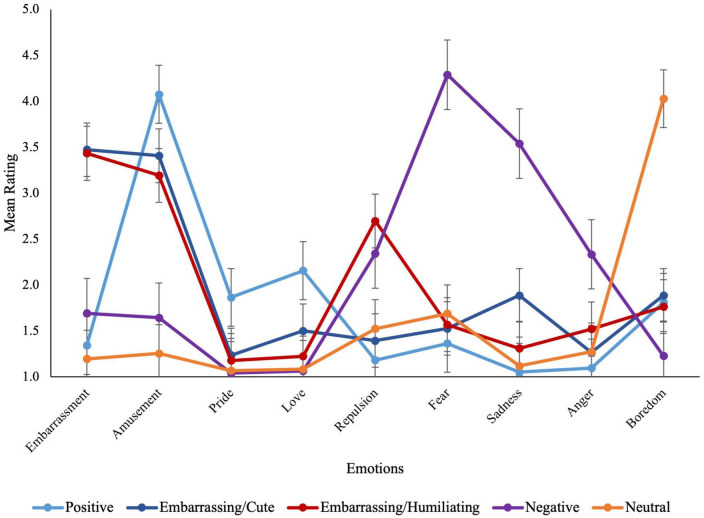
Mean emotion ratings for each of the measured emotions across film types.

Follow-up tests of simple main effects were used to explore the interactions; unless otherwise indicated, the contrasts described below are significant with Bonferroni correction applied. As seen in Figure 1, the positive films elicited the highest ratings of amusement, pride, and love (the positive emotions), and the lowest ratings of repulsion, fear, sadness, and anger, of any film type. Mixed/embarrassing and mixed/humiliating films produced similar ratings to one another in terms of pride, fear, boredom, and embarrassment (*p* > 0.05); however, whereas the mixed/embarrassing films elicited higher levels of amusement, love, and sadness than the mixed/humiliating films, the mixed/humiliating films elicited higher levels of repulsion and anger. Negative films produced the highest ratings of fear, sadness, and anger, and the lowest ratings of boredom across all film types. Finally, neutral films produced the lowest levels of amusement and embarrassment and the highest ratings of boredom of all film types. Taken together, these findings demonstrate that the five film types triggered distinctly different emotions in viewers, which were appropriate to the context.

Amusement, fear, and boredom were the strongest or “primary” emotions elicited by the positive, negative, and neutral films, respectively. The primary emotions elicited by both the mixed/embarrassing and mixed/humiliating film types were amusement and embarrassment. As a result, ratings of these emotions were averaged to create a composite measure (see Table 2 for mean ratings for each of the primary emotions).

**TABLE 2:** Mean ratings for the primary emotion and mean dispersion score for each of the five film types.

Film type	Strongest (primary) emotion or blend	Mean rating for strongest (primary) emotion (SD indicated)	Mean number of discrete emotions experienced (dispersion; SD indicated)
Positive	Amusement	4.08 (1.27)	3.95 (2.01)
Mixed/embarrassing	Amusement-embarrassment^a^	3.47 (0.95)	4.80 (2.19)
Mixed/humiliating	Amusement-embarrassment^a^	3.34 (1.02)	4.87 (2.04)
Negative	Fear	4.29 (1.44)	4.54 (1.74)
Neutral	Boredom	4.03 (1.43)	2.97 (2.03)

^a^As the mean ratings for these two emotions were equally high they were averaged to generate a composite score.

### 3.4. Assessing unique variance in measures from the behavioral task accounted for by SPS, alexithymia, childhood emotional abuse, and depression

#### 3.4.1. Predictors of the primary emotion elicited by each film type

Significant path coefficients for the MMR conducted to assess relationships between the TAS-20, HSPS, OS-ATQ, PHQ-9, and CTQ scores (predictors) and the primary emotion for each of the five film types (outcome variables) are presented in Table 3. The HSPS mean score was a significant positive predictor of Amusement ratings for the positive films and Fear ratings for the negative films, and the OS-ATQ mean score was a significant positive predictor of average Amusement-Embarrassment ratings for the mixed/embarrassing and mixed/humiliating films. Finally, the PHQ-9 total score was a significant negative predictor of Amusement ratings for the positive films. TAS-20 and CTQ scores did not significantly predict primary emotion ratings when controlling for SPS and current depressed mood.

**TABLE 3:** Significant path coefficients for multivariate multiple regressions.

Outcome variable	Film type	Predictor			
		**HSPS**	**OS-ATQ**	**TAS-20**	**PHQ-9**
		**B [95% CI]**	**B [95% CI]**	**B [95% CI]**	**B [95% CI]**
Strength of primary emotion	Positive	0.26 [0.04, 0.46]			−0.03 [−0.06, −0.00]
	Negative	0.41 [0.21, 0.64]			
	Mixed/embarrassing		0.16 [0.02, 0.29]		
	Mixed/humiliating		0.25 [0.11, 0.39]		
Dispersion	Positive	0.41 [0.11, 0.70]		0.02 [0.00, 0.04]	
	Negative			0.03 [0.01, 0.05]	
	Mixed/embarrassing	0.37 [0.03, 0.70]		0.04 [0.02, 0.06]	−0.05 [−0.10, −0.01]
	Mixed/humiliating	0.37 [0.11, 0.66]		0.04 [0.02, 0.06]	−0.05 [−0.09, −0.01]
	Neutral	0.28 [0.03, 0.57]		0.03 [0.01, 0.05]	
Feelings match	Positive	0.27 [0.08, 0.46]			
	Negative	0.41 [0.20, 0.62]			
	Mixed/embarrassing		0.29 [0.12, 0.46]		
	Mixed/humiliating	0.22 [0.07, 0.36]			
IRI/EI	Factor 1	0.50 [0.37, 0.63]	0.24 [0.12, 0.36]		
	Factor 2	0.30 [0.17, 0.41]	0.31 [0.19, 0.42]	−0.01 [−0.02, −0.01]	

Shown are significant regression (B) coefficients and bias-corrected accelerated 95% confidence intervals (CIs) calculated using 1000 bootstrapped samples. HSPS, Highly Sensitive Person Scale; OS-ATQ, Orienting Sensitivity subscale from the Adult Temperament Questionnaire; TAS-20, Toronto Alexithymia Scale; PHQ-9, Patient Health Questionnaire; IRI/EI, Interpersonal Reactivity Index/Empathy Index.

#### 3.4.2. Predictors of dispersion in the emotions elicited by each film type

For each participant, a “dispersion” score was computed for each film type by counting the number of emotions that received a mean rating across the two films of a given type that was higher than 1 (do not agree at all). This count could range from zero (neither film of a given type elicited a rating >1 for any emotion) to nine (both films of a given type elicited a rating >1 for every emotion). This score indicated how varied the emotions elicited by each film type were, with higher scores indicating greater dispersion. As expected, a repeated-measures ANOVA confirmed that the mixed-valence films produced the most varied reactions. After Bonferroni’s correction, the only contrasts that were *not* significantly different were those between the negative and mixed/embarrassing films, and between the mixed/embarrassing and mixed/humiliating films (see Table 2).

Significant path coefficients for the MMR conducted to assess the relationships between the TAS-20, HSPS, OS-ATQ, CTQ, and PHQ-9 scores (predictors) and the dispersion scores for each film type (outcome variables) are shown in Table 3. The TAS-20 total score was a significant positive predictor of dispersion scores for all film types. The HSPS mean score positively predicted dispersion scores for the positive, mixed/embarrassing, mixed/humiliating, and neutral film types. The PHQ-9 total score negatively predicted dispersion scores for the mixed/embarrassing and mixed/humiliating films. OS-ATQ and CTQ scores did not significantly predict dispersion scores when holding other predictors constant.

#### 3.4.3. Predictors of the perceived match between emotions experienced by oneself and by the person featured in films of a given type

After rating how strongly they felt each emotion while watching a film clip, participants rated how closely the emotions they experienced matched those experienced by the main person featured in the film clip. This question was intended to tap into the other-focused component of empathy. The mean “match” ratings for the five film types were entered as outcome variables in an MMR, with the TAS-20, HSPS, OS-ATQ, CTQ, and PHQ-9 scores entered as predictor variables.

As seen in Table 3, HSPS mean scores positively predicted match ratings for positive, negative, and mixed/humiliating films, and OS-ATQ mean scores positively predicted match ratings for mixed/embarrassing film types. The TAS-20, CTQ, and PHQ-9 scores did not significantly predict these ratings when holding all other predictors constant. This pattern of results closely parallels that seen in the model predicting the strength of the primary emotion(s) experienced while viewing each film type. Indeed, primary emotion and match scores for corresponding film types were positively correlated, with moderate-to-large effect sizes [0.29 ≤ *r*(305) ≤ 0.68, *p* < 0.001]; the exception was the correlation between boredom ratings and match ratings for the neutral films, which was somewhat weaker but still significant [*r*(305) ≤ 0.17, *p* = 0.003]. Thus, individuals who reported feeling the primary emotion(s) more strongly were also more likely to infer that their feelings mirrored those of the person featured in the film.

### 3.5. Predicting empathy-related processes assessed through self-report

Using exploratory factor analysis, we replicated the two-factor structure of the IRI/EI subscales found by Jordan et al. (2016; factor loadings can be found in the Supplementary material).

Factor 1, including the PD, EMP and BC subscales, was termed the *self-oriented* factor, as items within these subscales require the individual to reflect on their awareness of how certain situations and others’ emotions and behaviors affect them personally. Factor 2, including the EC and PT subscales, was termed the *other-oriented* factor, as these subscales address an individual’s ability to adopt another’s perspective and feel concern for them.

Significant path coefficients for the MMR conducted to assess relationships between the TAS-20, HSPS, OS-ATQ, CTQ, and PHQ-9 scores (predictors) and the two IRI/EI factor scores (outcome variables) are presented in Table 3. HSPS and the OS-ATQ mean scores positively predicted both factor scores, whereas the TAS-20 total score negatively predicted only the other-oriented factor score. The CTQ and PHQ-9 did not significantly predict either factor score when holding other predictors constant.

### 3.6. Associations between IRI/EI measures and performance on the behavioral task

Collapsing across the five film types, we computed the average strength of the primary emotion(s) experienced during film viewing, the mean dispersion score, and the mean match rating for each participant. Supplementary analyses confirmed that the relationships described above were still captured with these mean scores. Thus, HSPS and OS-ATQ scores positively predicted the mean primary emotion ratings; TAS-20 and HSPS scores positively predicted, and PHQ-9 scores negatively predicted, the mean dispersion scores; and HSPS and OS-ATQ scores positively predicted the mean match ratings (see Supplementary material). Having established this, we then ran correlational analyses to examine relationships between the two IRI/EI factor scores and the mean scores from the behavioral task.

As seen in Table 4, the three behavioral task variables were significantly correlated with one another (with small-to-large effect sizes), as were the two IRI/EI factor scores (with a large effect size). More importantly, whereas scores on the self-oriented factor (Factor 1) were significantly correlated with all three of the behavioral task variables, scores on the other-oriented factor (Factor 2) were significantly correlated with the mean primary emotion and match ratings but not with the mean dispersion score. Although the effect sizes were small, these latter findings support the idea that performance on our behavioral task was related to participants’ personal views about their empathic abilities, as conveyed through self-report. They also suggest, however, that the *range* of feelings one experiences during emotional contagion contributes more strongly to self-oriented processes, such as those that generate feelings of personal distress, than to other-oriented processes, such as those underlying perspective taking and empathic concern.

**TABLE 4:** Pearson correlation coefficients measuring the relationships between the behavioral task measures and the IRI/EI factors.

	Mean primary emotion	Mean dispersion	Mean match	Factor 1 self-oriented	Factor 2 other-oriented
Mean primary emotion	–				
Mean dispersion	0.41**	–			
Mean match	0.56**	0.23**	–		
Factor 1	0.24**	0.23**	0.25**	–	
Factor 2	0.22**	0.06	0.20**	0.66**	–

IRI/EI, Interpersonal Reactivity Index/Empathy Index.

**Correlation is significant at the 0.01 level (2-tailed).

## 4. Discussion

The key objective of the present study was to tease apart variance in emotional contagion and other empathy-related constructs accounted for by alexithymia and SPS. This was important as these traits are commonly studied separately despite the fact that they are positively associated with one another (Liss et al., 2008; Jakobson and Rigby, 2021; Karaca Dinç et al., 2021), and that aspects of both traits predict how we react to emotional scenes (Rigby et al., 2020). We also controlled for two potentially relevant variables in our analyses: childhood emotional abuse and current depressed mood. This was warranted because childhood adversity is associated with higher levels of alexithymia (e.g., Bermond et al., 2008) and greater functional impairment in those with SPS (Aron et al., 2012); and all three of these variables are associated with depression (e.g., Deng et al., 2006; Jakobson and Rigby, 2021), which itself has been linked to reduced emotional reactivity (Rottenberg et al., 2002) and impaired affective empathy (Yan et al., 2021).

### 4.1. Prediction of emotion contagion and other processes related to empathy

Research exploring relationships between SPS and self-report measures of empathy is very limited (e.g., Schaefer et al., 2022). One might expect a positive association given that SPS is associated with in-depth processing of self and other emotional states, and given the fact that neuroimaging research has identified increased neural activity in individuals with SPS in regions associated with empathy, self-other awareness, and self-referential processing during emotional processing tasks (Acevedo et al., 2018). Indeed, a positive association was supported in the current study, with SPS predicting higher levels of both self- and other-oriented processes related to empathy. The results from our behavioral task suggest that strong emotional contagion and self-other awareness may partially explain these effects. We observed that SPS positively predicted how strongly participants felt the primary emotion elicited by each film clip, the range of emotions that were experienced, and the extent to which an individual’s personal emotional responses were perceived to match those of the person featured in the film clips. The strength of the primary emotions would make them easily discernable. Attending to and carefully analyzing both the primary and any additional elicited emotions may help those with SPS generate a nuanced and deeper appreciation of their own reactions and this, in turn, may help them to make inferences about how others are feeling and facilitate the emotion matching process. Although our results suggest that individuals with SPS view themselves as empathic, it remains to be seen whether they actually engage in more prosocial behavior in real-world situations. Given that self-oriented processes negatively predict prosocial action (Jordan et al., 2016), it may be important to consider the *relative* strengths of self- and other-oriented processes when attempting to predict how individuals with SPS will behave outside the laboratory.

As expected, alexithymia negatively predicted other-oriented processes related to empathy, a result that is in line with previous findings (e.g., Brett and Maybery, 2022). Although positive correlations were seen between alexithymia and two of the self-oriented subscales of the IRI/EI (PD and EMP), alexithymia did not significantly predict the self-oriented factor score when taking SPS, childhood emotional abuse, and current depressed mood into account. It did, however, positively predict the number of discrete emotions elicited by the films (i.e., dispersion). It may be that, in individuals with alexithymia, the extent to which one turns one’s attention inward and *examines* one’s emotions is an important determinant of the nature of one’s empathic responses. This tendency can vary widely; indeed, Jakobson and Rigby (2021) suggested that there may be two subtypes of alexithymia distinguished, in part, on this basis. In those who score high on externally oriented thinking (i.e., who have a strong external focus), secondary emotions might simply create “noise” that interferes with the ability to identify and describe one’s feelings precisely. In those scoring lower on this subscale, who are better able to turn their attention inward and whom Jakobson and Rigby (2021) described as being more highly sensitive, experiencing a diffuse set of strong emotional reactions might be expected to exacerbate feelings of personal distress. In either case, other-oriented processes would be negatively impacted. This, of course, can have real-life consequences. For instance, Patil and Silani (2014) have argued that, because alexithymic individuals show reduced concern for others, they are more likely to feel that “accidental” harms (i.e., those committed by someone who does not *believe* they are causing harm) are morally acceptable; in other words, their moral judgments are more lenient than those of individuals scoring low on alexithymia. Along similar lines, Zhang et al. (2020) found that individuals scoring high in alexithymia were more likely to make utilitarian than deontological judgments (i.e., to conclude that “the ends justify the means”).

When controlling for other variables, depression symptom severity did not significantly predict how strongly participants experienced the primary emotions elicited by the films (except for feelings of amusement when watching the positive films), the sense that their feelings matched those of the people featured in the films, or self- or other-oriented factor scores. Interestingly, depression symptom severity did negatively predict dispersion scores for the mixed-valence (mixed/embarrassing and mixed/humiliating) films when taking all other variables into account. Thus, individuals reporting stronger symptoms of depression were less likely to feel a multitude of emotions after watching these films and were less likely to experience positive emotions when watching positive films. Others have also reported that individuals with depression experience decreased positive and negative emotions when viewing emotional stimuli (Rottenberg et al., 2002). This could be related to top-down emotional suppression, which is strongly associated with depression (e.g., Dawel et al., 2021). Interestingly, emotional suppression while watching emotional film clips has been associated with a poorer recovery from depression (Rottenberg et al., 2002).

### 4.2. Emotional abuse in childhood and its relationship to empathy-related processes and depression

In the current study, scores on the childhood emotional abuse measure did not significantly predict performance on the behavioral task or factor scores on the IRI/EI, after controlling for other variables. Greenberg et al. (2018) reported that individuals who had experienced childhood trauma showed elevated levels of empathy as adults—particularly regarding other-oriented processes such as showing empathic concern. Importantly, however, these researchers did not control for SPS in their study. Given that SPS was positively associated with both childhood emotional abuse and other-oriented processes related to empathy in our study, it is possible that the relationship that Greenberg et al. (2018) described was mediated by SPS.

Consistent with past research (e.g., Zhang et al., 2020), we did observe that childhood emotional abuse was positively associated with depression in the current study. The results from a recent study by Karaca Dinç et al. (2021) suggest that both alexithymia and SPS may mediate this link. One potential explanation for mediation via alexithymia is that a lack of affect sharing and mirroring between caregiver and child contributes to difficulties with identifying and describing affective states later in life (Xie et al., 2021), and that these problems contribute to emotional dysregulation and depressed mood. In the current study, individuals with higher levels of alexithymia experienced a large number of discrete emotions in response to emotional films, even after controlling for childhood emotional abuse and current depressed mood. Mediation via SPS may occur because those scoring high on this trait are particularly sensitive to adverse developmental environments (Aron and Aron, 1997; Liss et al., 2008; Aron et al., 2012). Aron et al. (2005) identified a strong causal effect of an adverse childhood environment on negative affectivity in adulthood for individuals with SPS. These authors proposed that the increased depth of processing that these individuals engage in (specifically concerning their social and emotional experiences) is what leads them to be especially affected by early adversity. They argue, however, that individuals with SPS who have not experienced early adversity are no more likely to experience negative affectivity than individuals without SPS (Aron and Aron, 1997; Aron et al., 2005).

### 4.3. Implications

This study makes a novel contribution by assessing the unique contributions that SPS and alexithymia make to the prediction of empathy-related processes assessed through both self-report and behavioral measures. Expanding our knowledge in this area could open new lines of research aimed at investigating the impact having high or low levels of empathy can have on socioemotional functioning. Deficits in empathy have been identified in certain populations, such as individuals with autism spectrum disorder, psychopathy, or antisocial, borderline, or narcissistic personality disorders (Decety and Moriguchi, 2007), and these deficits can contribute significantly to interpersonal difficulties. However, experiencing high levels of empathy or personal distress (e.g., during periods of conflict) can also be detrimental (Zurek and Scheithauer, 2017).

Research like that described in the current study may inform the continued refinement of targeted treatments for those with empathy differences and associated emotional regulation difficulties. Our findings suggest that individuals with SPS who experience strong emotional contagion run the risk of experiencing high levels of personal distress that could overwhelm them emotionally. Empathy-related psychological treatment for such individuals should focus on building emotional regulation and distress tolerance skills (Acevedo, 2020). Individuals with alexithymia in the current study experienced difficulties differentiating between their own emotional states, potentially leading to difficulties identifying the emotional states of others. Although there is currently no “gold-standard” treatment for alexithymia, in their review of psychological interventions targeting alexithymia, Cameron et al. (2014) found that successful treatments consistently utilized psychoeducation and skills training to improve emotional awareness. Such approaches may also prove useful in reinforcing the self-other distinction that is necessary for empathy. Indeed, Saito et al. (2016) found that participants with alexithymia were better able to correctly estimate the pain another experienced in a particular body part after being instructed to identify the body part as belonging to that person.

### 4.4. Limitations and future directions

Due to the COVID-19 pandemic, this study was completed online and remotely. As such, the size and resolution of the emotional film clips could not be standardized across participants. A second limitation was that most of the participants were female. Some studies have identified sex differences in relation to self-oriented processes related to empathy (e.g., Goerlich-Dobre et al., 2015), and so further work should be undertaken to determine if the results of the current study apply across both sexes, or are relevant to females only. It is also possible that other sociodemographic, psychological, and/or clinical variables implicated in emotion regulation and recognition processes that were not controlled for here affected our results.

We relied exclusively on self-report measures to assess alexithymia and SPS. Although this could be viewed as a limitation, we would point out that: (a) the measure of alexithymia that we employed is the most widely used measure of this construct world-wide and is valid and reliable (Bagby et al., 2020); and (b) following the advice of Aron et al. (2012), we supplemented the HSPS with the OS-ATQ to obtain a more fulsome assessment of SPS. The latter decision proved to be important, as scores on the OS-ATQ (which taps into how strongly we attend to both subtle external events and our own thoughts and mental images) were found to account for unique variance in the prediction of responses to mixed valence films.

Of course, our behavioral task required participants to reflect on their conscious experience of their emotional reactions to affective film clips. As such, the ratings participants gave, like their responses on the self-report measures, were clearly subjective. This may have made it difficult for those with strong alexithymic tendencies to provide valid and reliable responses. Given this, it would be interesting in future research to extend this work by collecting neuroimaging measures during the behavioral task, in addition to obtaining subjective reports. Empathy is associated with activity in specific neural regions and resting-state networks, and both alexithymia and SPS have been associated with altered activity in specific neural regions required for empathy (Bird et al., 2010; Goerlich-Dobre et al., 2015; Acevedo et al., 2018). Obtaining psychophysiological measurements would also provide novel data about the relationships between empathy, alexithymia, and SPS. Alexithymia has been associated with differences in heart rate variability, skin conductance response, and electromyography during empathy tasks (Sonnby-Borgström, 2009; Bogdanov et al., 2013; Cecchetto et al., 2018; Lischke et al., 2018; Härtwig et al., 2020). Conducting both neuroimaging and psychophysiological investigations would serve as a catalyst for future research into individual differences in processes like emotional contagion, which support empathy and prosocial behavior.

Finally, the focus of the current study was intended to be on the developmental form of alexithymia seen in the general population; as such, we restricted participation to individuals with no reported history of neurological disorders or significant head injuries. Future researchers could extend this by investigating emotional contagion in individuals with acquired forms of alexithymia, which occur at high rates in a range of neurological disorders, including traumatic brain injury and Parkinson’s disease (Hogeveen and Grafman, 2021).

## 5. Conclusion

The results of this study provide new insights into factors that contribute to individual differences in key socioemotional processes. We took the novel approach of assessing unique variance associated with alexithymia, SPS, childhood emotional abuse, and depression in the prediction of both emotional contagion assessed in a behavioral task and empathy-related processes assessed through self-report. Alexithymia predicted deficits in other-oriented processes (such as perspective-taking and empathic concern). We suggested that these deficits may arise, in part, because individuals scoring high on this trait fail to attend to and correctly interpret their mixed emotional reactions to others. In contrast, SPS predicted stronger self- and other-oriented processes related to empathy, possibly because those scoring high on this trait are very aware of their strong, nuanced emotional responses to other people. These observations are important for theory building, but they may also inform efforts to identify and support those experiencing atypical emotional awareness and socioemotional difficulties.

## Data availability statement

The dataset for this study can be found in the “Replication Data for: Individual differences in emotional contagion and empathy” repository https://doi.org/10.34990/FK2/XA88II.

## Ethics statement

The studies involving human participants were reviewed and approved by the Research Ethics Board, University of Manitoba Fort Garry Campus. The patients/participants provided their written informed consent to participate in this study.

## Author contributions

AM, SS, and LJ developed the research and analysis plan and wrote the manuscript. AM collected and analyzed the data. All authors contributed to the article and approved the submitted version.
