# Supplementary material for: Alexithymia and sensory processing sensitivity account for unique variance in the prediction of emotional contagion and empathy
Source: Front Psychol. 2023 Apr 20;14:1072783. doi: 10.3389/fpsyg.2023.1072783 (PMC10157231; doi:10.3389/fpsyg.2023.1072783)
Supplement: Supplementary file 1 [file Data_Sheet_1.docx]

Supplementary Materials

# Supplementary Tables

**SUPPLEMENTARY TABLE 1.** *Film Clips Included in the Emotional Contagion Behavioural Task*

| Valence | Film description | Number of individuals |
| --- | --- | --- |
| Positive | A baby dancing at a wedding. | Group |
| Positive | A person spins on the handrails of an escalator. | Single |
| Mixed/embarrassing | A person trying to leave a building crashes into a glass door. | Single |
| Mixed/embarrassing | A wedding photographer walking backwards while taking pictures falls into a fountain. | Group |
| Mixed/humiliating | A bride loses her fake teeth while drinking. | Group |
| Mixed/humiliating | An employee tries to photocopy his behind and breaks the glass on the photocopier. | Single |
| Negative | A boy jumps out of a car and is hit by another oncoming car. | Single |
| Negative | A bull attacks a torero while people watch. | Group |
| Neutral | A person sits and drinks tea. | Single |
| Neutral | View of a busy street with cars and pedestrians. | Group |

*Note:* The film clips and above information are from a collection compiled by Samson et al. (2016).

**SUPPLEMENTARY TABLE 2.** *Significant Path Coefficients* *between Predictor Variables and Mean Primary Emotion, Mean Match Scores, and Mean Dispersion Scores Across Film Types*

| Outcome Variable | Predictor | | | |
| --- | --- | --- | --- | --- |
|  | HSPS | OS-ATQ | TAS-20 | PHQ-9 |
|  | B [95% CI] | B [95% CI] | B [95% CI] | B [95% CI] |
| Mean Primary Emotion | .15 [.03, .28] | .14 [.01, .26] |  |  |
| Mean Feelings Match | .21 [.09, .34] | .14 [.01, .26] |  |  |
| Mean Dispersion | .31 [.08, .54] |  | .03 [.02, .05] | -.04 [-.07, -.00] |
| *Note*: Shown are significant regression (B) coefficients and bias-corrected accelerated 95% confidence intervals (CIs) calculated using 1000 bootstrapped samples. HSPS = Highly Sensitive Person Scale; OS-ATQ = Orienting Sensitivity subscale from the Adult Temperament Questionnaire; TAS-20 = Toronto Alexithymia Scale; PHQ-9 = Patient Health Questionnaire; IRI/EI = Interpersonal Reactivity Index/Empathy Index. | | | | |

**SUPPLEMENTARY TABLE 3.** *Factor Loadings of the IRI/EI Subscales*

| Subscale | Factor 1  Self-Oriented | Factor 2  Other-Oriented |
| --- | --- | --- |
| EMP | 0.85 |  |
| BC | 0.63 |  |
| PD | 0.48 |  |
| PT |  | 0.83 |
| EC |  | 0.50 |

*Note:* A cut-off factor score of 0.35 was used. Subscales of the Interpersonal Reactivity Index/Empathy Index (IRI/EI): EMP = Empathy; BC = Behavior Contagion; PD = Personal Distress; PT = Perspective Taking; EC = Empathic Concern.
